# Effects of food abundance and early clutch predation on reproductive timing in a high Arctic shorebird exposed to advancements in arthropod abundance

**DOI:** 10.1002/ece3.2361

**Published:** 2016-09-23

**Authors:** Jeroen Reneerkens, Niels Martin Schmidt, Olivier Gilg, Jannik Hansen, Lars Holst Hansen, Jérôme Moreau, Theunis Piersma

**Affiliations:** ^1^ Conservation Ecology Group Groningen Institute for Evolutionary Life Sciences (GELIFES) University of Groningen Groningen The Netherlands; ^2^ Arctic Research Centre Department of Bioscience Aarhus University Roskilde Denmark; ^3^ Laboratoire Biogéosciences Université de Bourgogne Dijon France; ^4^ Groupe de Recherche en Ecologie Arctique (GREA) Francheville France; ^5^ NIOZ Royal Netherlands Institute for Sea Research Department of Coastal Systems and Utrecht University Den Burg Texel The Netherlands

**Keywords:** Bird migration, *Calidris alba*, chick growth, climate change, nest survival, phenology, timing, trophic interactions, trophic mismatch

## Abstract

Climate change may influence the phenology of organisms unequally across trophic levels and thus lead to phenological mismatches between predators and prey. In cases where prey availability peaks before reproducing predators reach maximal prey demand, any negative fitness consequences would selectively favor resynchronization by earlier starts of the reproductive activities of the predators. At a study site in northeast Greenland, over a period of 17 years, the median emergence of the invertebrate prey of Sanderling *Calidris alba* advanced with 1.27 days per year. Yet, over the same period Sanderling did not advance hatching date. Thus, Sanderlings increasingly hatched after their prey was maximally abundant. Surprisingly, the phenological mismatches did not affect chick growth, but the interaction of the annual width and height of the peak in food abundance did. Chicks grew especially better in years when the food peak was broad. Sanderling clutches were most likely to be depredated early in the season, which should delay reproduction. We propose that high early clutch predation may favor a later reproductive timing. Additionally, our data suggest that in most years food was still abundant after the median date of emergence, which may explain why Sanderlings did not advance breeding along with the advances in arthropod phenology.

## Introduction

As a consequence of higher spring temperatures, many organisms have advanced their phenology (Post et al. 2001; Root et al. 2003). Many bird populations, for example, have started to migrate and breed earlier in association with increasing spring temperatures (Both et al. 2005; Gordo 2007; Lehikoinen and Sparks 2010) and advancements in snow melt (e.g., Liebezeit et al. 2014). Organisms at higher trophic levels often advance less than those at lower trophic levels (Both et al. 2009; Thackeray et al. 2010), resulting in a temporal uncoupling of trophic interactions (Parmesan and Yohe 2003; Post and Forchhammer 2008). Phenological mismatches can have negative fitness consequences (Miller‐Rushing et al. 2010) and may in migratory birds lead to population declines (Both et al. 2006; Møller et al. 2008; Saino et al. 2011; Dunn and Møller 2014).

Several mechanisms have been proposed to explain mismatches between the timing of avian reproduction and prey availability. A rigid timing of avian seasonal events such as migration and reproduction may constrain responses (Both and Visser 2001; Knudsen et al. 2011). Long‐distance migratory birds time the onset of migration with limited knowledge of the ecological conditions in the distant breeding area later in the year (Piersma et al. 1990; Visser et al. 2006; Winkler et al. 2014). A hurdle in making adjustments includes the problem that, whereas photoperiodic cues used to time migration (Gwinner 1996a,b) are unaffected by climate change (Coppack et al. 2003), the timing of peak abundance of arthropod prey is strongly so (e.g., Høye and Forchhammer 2008; see Winkler et al. 2014 for discussion). Environmental conditions during migration may also constrain the possibility to arrive earlier on the breeding grounds (e.g., Piersma and Baker 2000; Both 2010).

The climate in the Arctic is changing faster than in any other region on Earth, with temperature increases nearly twice the pace as the global average (e.g., McBean et al. 2005; Pithan and Mauritsen 2014). This has major implications for Arctic vertebrate species and communities (Meltofte et al. 2008a; Post et al. 2009; van Gils et al. 2016). However, with few exceptions to do with Arctic geese (e.g., Dickey et al. 2008; Gauthier et al. 2013), studies of the consequences of phenological mismatches in birds have largely focused on the temperate zone (Møller et al. 2006). The available studies of Arctic shorebirds mostly demonstrated the effects of mismatches indirectly, either using climate model predictions (e.g., Tulp and Schekkerman 2008; Pearce‐Higgins et al. 2010), or by showing spring advancement of arthropods and their predators, but without directly documenting ecological and/or fitness consequences of possible phenological mismatches (Høye et al. 2007; van Gils et al. 2016; but see McKinnon et al. 2012; Senner et al. 2016).

An optimal timing of reproduction is usually explained by seasonal changes in the availability of food (Lack 1950; Perrins 1969). Individuals that raise young during the peak in food abundance often perform better than individuals that breed later (e.g., Verhulst et al. 1995; Brudney et al. 2013). Shifts in the peak of species’ food abundance relative to the shift in the phenological event (e.g., migration or reproduction) increase the selective pressure to change breeding accordingly, and failure to do so is often used as a simple yardstick to assess the extent of the mismatch. However, this focuses on the timing of food peaks as the only selective pressure on phenology (Visser and Both 2005; Jonzén et al. 2007) and ignores the level of abundance of the food source (Durant et al. 2005). Yet, food availability is one of several selective pressures that may shape the optimal timing of reproduction (Drent and Daan 1980).

Clutch predation is an important source of reproductive failure in birds (Ricklefs 1969; Macdonald and Bolton 2008), particularly in the Arctic (e.g., McKinnon et al. 2010). If the risk of clutch predation varies intraseasonally (Smith and Wilson 2010), clutch predation could additionally constrain or favor phenological change of avian reproduction in step with their arthropod prey (Dunn and Winkler 2010; Schmidt et al. 2015). To date, such top‐down selective pressures by organisms at higher trophic levels that feed upon birds and their offspring have yet to be examined as a factor that may limit a forward shift in the timing of breeding (Both et al. 2009). For noncolonial, ground‐breeding Arctic birds, predation risk of the clutches may depend on the density of nests and be related to the extent of snow cover (Byrkjedal 1980). Snow cover varies between years and has large consequences on the timing of breeding of Arctic shorebirds (Green et al. 1977; Meltofte et al. 2008b; Smith et al. 2010). As spring progresses, the extent of tundra covered with snow decreases rapidly and the number of bird clutches gets “diluted” in a larger snow‐free area, making it increasingly difficult for foxes to detect them (see Seymour et al. 2004).

Using a dataset collected during a 17‐year period in northeast Greenland, a period with rapid warming (e.g., Høye et al. 2013), we investigated the inter‐ and intra‐annual variation in, and the interlinkages between the reproductive timing of a long‐distance migratory high Arctic bird, the Sanderling (*Calidris alba* Pallas 1764, Fig. 1), clutch predation, chick growth, and arthropod prey abundance. We hypothesize that clutches have a larger risk to be found by predators early in the season, especially in snow‐rich years, and that Sanderling chicks grow better if they hatched just before or during the peak abundance of their arthropod prey.

**Figure 1:**
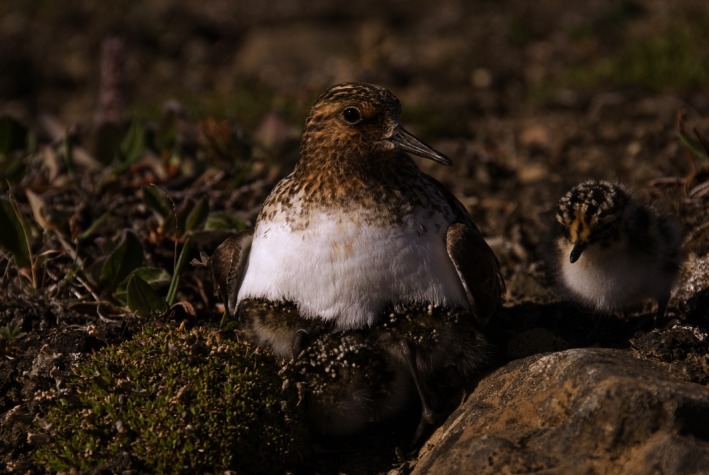
An adult Sanderling (*Calidris alba*) incubating a full brood of four chicks at Zackenberg, northeast Greenland (Photograph by Jeroen Reneerkens).

## Materials and Methods

### Study species

Sanderlings are long‐distance migratory sandpipers. The coastal nonbreeding area of the Greenlandic population ranges from Namibia in the south and northern Scotland in the north (Conklin et al. 2016; Loonstra et al. 2016). Typically, Sanderlings arrive at their breeding grounds in northeast Greenland between late May and mid‐June (Meltofte et al. 2007b). Like most sandpipers (Sandercock 1997), the clutch size in Sanderlings is four eggs, but occasionally smaller clutch sizes occur (Piersma et al. 1996). Eggs are laid in a small scrape lined with leaves. After the third egg has been laid, birds start irregular incubation and after the clutch is completed the eggs are intermittently incubated. Egg laying takes 4 days and incubation takes an additional 22 days, as with most other sandpipers (Piersma et al. 1996). When a clutch is depredated, sometimes a replacement clutch is laid, but usually not later than 1 July (Meltofte et al. 2007b; Reneerkens et al. 2014). Replacement clutches can be with either the same or a new partner in a nearby territory (Reneerkens et al. 2014). Within 24 h after hatch, the precocial chicks leave the nest cup as a family unit (or “brood”) guided by one of the parents. At an age of ca. 16 days, the chicks can make their first short flights.

### Study area

The study was conducted at Zackenberg (74°28′N 20°34′W). The study area in Zackenberg is a ca. 60 km^2^ large valley, with a focal census research area of 19 km^2^ in size, where various biotic and abiotic variables have been monitored in a standardized way since 1996 until recent (Meltofte et al. 2008a). We used additional data on Sanderling chicks from a nearby field site at Hochstetter Forland (75°10′N 19°45′W), located 80 km north of Zackenberg to construct a growth curve for the species.

The suspected main predator of shorebird eggs in northeast Greenland is the Arctic fox (*Vulpes lagopus* Linnaeus 1758). Long‐tailed skuas (*Stercorarius longicaudus* Vieillot 1819) may take eggs, but are more focused on chicks. Raven (*Corvus corax* Linnaeus 1758)*,* Glaucous gull (*Larus hyperboreus* Gunnerus, 1767), and Stoat (*Mustela erminea* Linnaeus 1758) are also known to prey on birds and their eggs, but they are less common in our study area and probably play little role in the predation of clutches and chicks.

### Seasonal abundance of arthropods

We used five plots of 10 × 20 m^2^ each containing eight yellow pitfall traps (10 cm diameter). From 2007 onwards, only four pitfalls within plots were used. Pitfalls were emptied weekly during June and July. All plots were operated during 1996–2013 except for 2010. One of the plots was closed in 1999. The plots are placed in wet fens and mesic heaths. *Eriophorum scheuchzeri* (Hoppe) dominated the wet fen, whereas *Salix arctica* (Pallas), *Cassiope tetragona* (Don), and *Dryas* sp. dominated the remaining plots (see Høye et al. 2013 for details about the arthropod collection with pitfalls).

We used the number of arthropod prey items collected in pitfalls as a combined measurement of arthropod abundance and their activity on the tundra surface (e.g., Reneerkens et al. 2011); a measure that is known to be closely related to growth of arctic shorebird chicks (Schekkerman et al. 2003; McKinnon et al. 2012). Biomass estimates of the collected arthropods were not available, but for a different set of arthropod collections using pitfalls in Zackenberg, we show that daily dry mass estimates and daily number of arthropods strongly correlate (*R* ^2^ = 0.74; Appendix S1).

We included all specimens in the orders of Aranea, Diptera, Hemiptera, Hymenoptera, and Lepidoptera (larvae and imagines), which have been shown to be part of the diet of Sanderlings in Zackenberg (Wirta et al. 2015). For each year, we estimated the date of median arthropod abundance, per arthropod order and for all specimens combined, by linear interpolation between the last date when <50% of the seasonal capture was reached and the first date when more than 50% of the seasonal capture was reached. In all years, the date of median arthropod abundance coincided with the date of maximum arthropod abundance (number of individuals per trap) within a year (i.e., “food peak height”). The “food peak width” is the period between the dates at which 25% and 75% of the arthropods were collected, and both the food peak height and the food peak width were used to describe the annual food abundance. We defined “mismatch” as the temporal difference (in days) between the hatch date of a chick and the median date of arthropod abundance.

### Nest searching and monitoring

We searched for Sanderling nests and broods by foot in Zackenberg in June and July 1996–2013. In case the exact laying date was unknown, we estimated it by determining clutch age by flotation of two eggs in each clutch (Liebezeit et al. 2007; Hansen et al. 2011). This also allowed us to predict the hatch date, and we always visited the clutches 2–3 days before expected hatch dates to check for signs of hatching (i.e., cracks or holes in the eggs) and to visit the nests at hatch to measure and ring the hatched chicks. In 2007–2013, we equipped the majority (157 of 194) of nests with small temperature loggers (Tiny Tag, Gemini) with a probe placed between the eggs. The temperature loggers collected temperature data every minute for a minimum of 22 days. If clutches were lost, the temperature profiles accurately indicated when this occurred (Reneerkens et al. 2011). Nests without temperature loggers were checked every 1–5 days until found depredated or until hatch. Clutches were considered successful if at least one chick hatched, and considered unsuccessful when temperature logger data indicated clutch loss, or when the nest was found empty before expected hatching. We considered clutches abandoned when cold eggs were encountered in the nest cup and the temperature profiles of the temperature loggers indicated that the clutch had not been incubated for more than 2 days. Abandoned nests (*n* = 13) or clutches which failed for other reasons than predation (*n* = 5) were not included in the analyses.

The nest cup of each depredated clutch was examined for signs that could indicate the predator species or type. The smell of urine or the presence of fresh Arctic fox feces inside or near the empty nest cup was taken as signs of predation by Arctic fox. Also, if temperature loggers were dug out of the ground and/or had been chewed on, we classified Arctic fox as the predator. Nearly, complete shells of eggs with big holes indicated that an avian predator had pecked the eggs.

### Snow cover

Snow cover on the mountain slope, where the majority of Sanderling nests were found, was estimated using automated cameras from ca. 480 m above sea level at the mountain Zackenberg. The percentage of snow covered area was computed from the orthorectified digital photos (Hinkler et al. 2002). The images were regularly taken since 1999 and daily since 2007. We used the date of 50% snow cover as a measure of snow phenology. The annual average snow cover during the breeding season and the actual daily snow cover were used as covariates in an analysis of daily clutch survival (next paragraph).

### Clutch survival analysis

To evaluate the inter‐ and intra‐annual trends in daily clutch survival, we used the nest survival model implemented in program MARK, version 7.1, which takes biases in the detection of successful and nonsuccessful clutches into account (Dinsmore et al. 2002). We only have detailed clutch information (i.e., appropriate sample sizes and daily monitoring of nest success by use of thermologgers) for the period 2007–2013, where data were obtained on an average of 31 clutches (range: 25–46) annually. We tested a limited number of models in which clutch survival varied both between and within years and included date and year as covariates in our models. Within years, we expected positive linear or quadratic seasonal changes in clutch survival. Due to the highly synchronized egg laying within years, clutch age and date (day of year) were strongly correlated (Pearson's *r* = 0.98) and thus confounded. We therefore chose to only use date in our models. We included the annual average snow cover during the period that nests were followed (Syear) and the amount of snow cover per date (Sdate) as covariates in our models.

### Chick growth

Throughout the prefledging period (0–17 days), we captured and recaptured each chick within a family and measured their body mass using electronic scales with an accuracy of 0.1 g. Growth data were fitted to both a Gompertz growth model, *M *= *A*·exp (−exp (−*k*·(*t *− *T*))), and a logistic growth model, *M *= *A/*(1 + exp (−*k*·(*t *− *T*))), typically used to describe avian growth (Starck and Ricklefs 1998). In these formulas, *M* is body mass (g), *A* is the asymptotic mass (g), *k* is the growth coefficient, *t* is the age of the chick, and *T is* the age at the point of inflection. Growth curves were fitted using maximum likelihood in the nlme package of R (Pinheiro et al. 2015). The growth curves were based on 310 individual chicks of which 40 were measured at two ages and one was measured three times. To control for pseudo‐replication caused by the multiple measurements of these individuals, these nonlinear mixed effect models included individual as a random effect. The three growth parameters were included as fixed effects. The fit of both models based on AIC_c_ was compared, and the better fitting model was chosen to describe the data.

Because the interindividual differences in body mass at hatch are small, we assumed that deviations from the predicted growth curve are caused by differences in growth rate. We used the residuals from the predicted growth curves to calculate the body mass relative to the age‐corrected average. These residuals, expressed as percentage difference from the average (hereafter, “chick growth” or “growth”), were used to describe individual chick's relative growth. At hatch, shorebird chicks have energy stores in their residual yolk sac (Starck and Ricklefs 1998) and stay in the nest cup for at least some hours. Therefore, only chicks older than 1 day were used to study effects of hatch date in relation to food abundance on growth. Of 74 chicks (older than 1 day and excluding chicks measured in 2010 when no arthropod data were available), data were available for analyses of growth. All individuals were captured and measured only once (average age = 6.5 days), but included chicks from the same families (family and year were initially included as random factors in the model, see “Statistical analyses”). Hatch date relative to the median date of food abundance (i.e., the individual mismatch) may affect chicks’ growth rate depending on the seasonal shape of the abundance of arthropods (i.e., both the season's maximum and the width of the seasonal abundance of arthropods). Therefore, we used the following model to look for effects of arthropod abundance on chick growth:

Chick growth ~ mismatch + food peak height + food peak width + two‐way and three‐way interactions.

### Statistical analyses

We selected either linear or quadratic functions to describe the changing phenology of Sanderling hatch date and arthropods and of snow cover. Models with the best fit based on their *F*‐values were selected to describe the data. For direct comparison of shifts in phenology between birds and arthropods, we also analyzed the linear relationship of arthropod phenology.

The clutch survival analysis was performed in program MARK (White and Burnham 1999). We scaled dates such that 10 June was day 1, the earliest date at which a clutch was found. In total, we considered 11 candidate models, for all of which we used a logit link function. Models were ranked with the corrected Akaike's information criterion for small samples (AIC_c_), and ΔAIC_c_ and Akaike weights (*w*_*i*_) were used to infer support for models in the candidate set (Burnham and Anderson 2002). Our candidate model set contained a mix of models with linear and quadratic terms and interaction terms. Therefore, we could not use model averaging to interpret parameter estimates. We discuss the results from the models that are substantially better supported than other models and have ΔAIC_c_'s of at least 2 less than other models (Burnham and Anderson 2002). Models with ΔAIC_c_ < 2, but with additional parameters to other strongly supported models, were not considered fitting the data well because model deviance is not reduced sufficiently to overcome the penalty of 2 AIC_c_ for the additional parameters (Arnold 2010). Goodness‐of‐fit tests are not available for nest survival models in MARK (Dinsmore et al. 2002).

The effects of food peak width and height on chick growth were analyzed with mixed‐effects models using the package lme4 in R (Bates et al. 2015) with family and year as random effects. All variables were centered around the mean. First, we checked for the effects of inclusion of the separate random effects by comparing AIC of models with or without the different random effects. Subsequently, we used model simplification by stepwise deletion of nonsignificant fixed effects, by first deleting nonsignificant random effects, then nonsignificant interactions and then nonsignificant main effects if they were not part of a significant interaction.

## Results

### Advancing phenology of birds and arthropods

**Figure 2:**
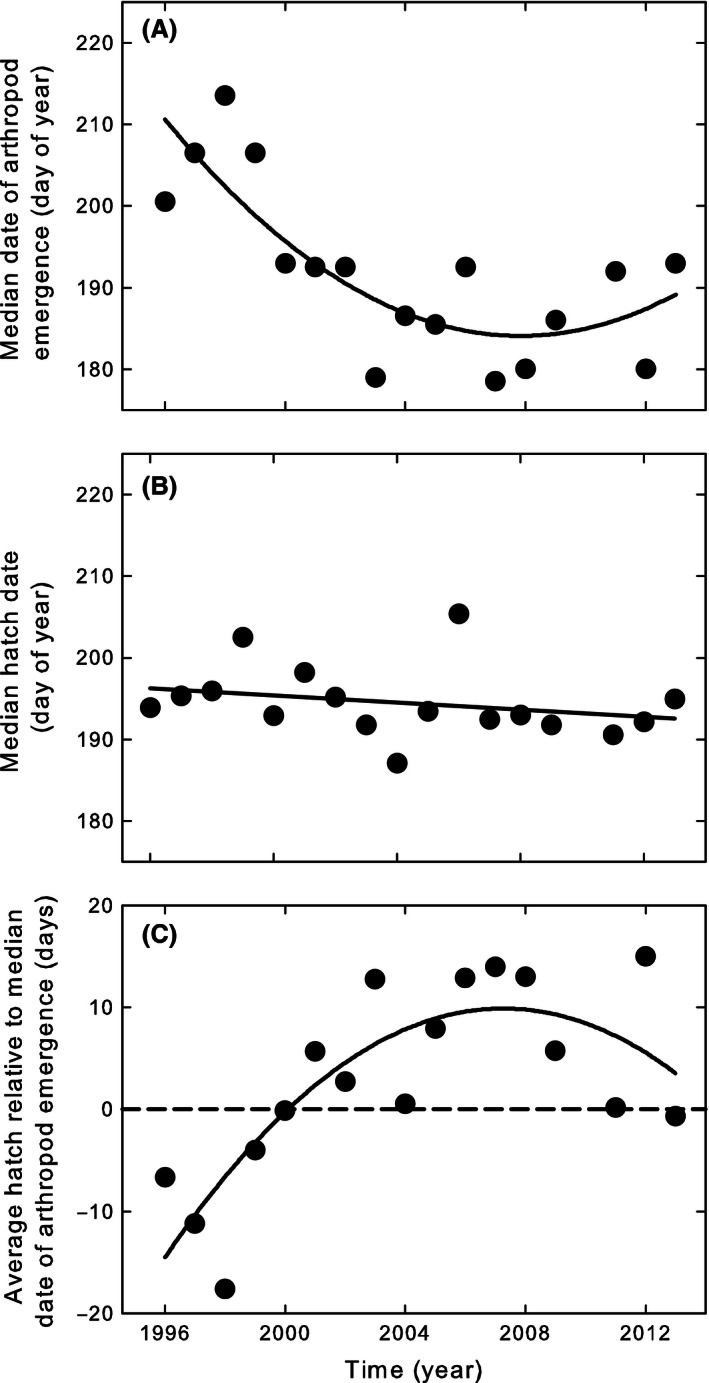
The date of (A) arthropod peak abundance (specimens of all arthropod orders combined) and (B) hatching of Sanderling advanced at different rates in Zackenberg 1996–2013. Consequently, the phenological mismatch between Sanderling and their prey has increased over time (C). The difference between average hatch date of Sanderling (B) and the median peak in arthropod peak abundance (A) resulted in phenological mismatches (C) since 2000. The dotted horizontal line in (C) indicates when Sanderling hatching and median arthropod peak abundance happened on the same date.

The date at which the snow cover of the tundra was reduced by half occurred increasingly early from 1999 to 2013, (approximately 1.79 days per year: regression, *F*_1,10_ = 8.1, *P* = 0.02, *R* ^2^ = 0.44). The temperature during our study period also increased (see supplementary material in Schmidt et al. 2016). In years with early snowmelt, the average hatch date of Sanderlings was also early, although this relationship was not statistically significant (linear regression, *F*_1,10_ = 4.5, *P* = 0.06). From 1996 to 2013, the median date of arthropod abundance advanced, but a quadratic function fitted the arthropod advancement better than a linear function, indicating that the advancement gradually levelled off (*F*_2,14_ = 12.8, *P* < 0.01, *R* ^2^ = 0.65; Fig. 2A). The linear advancement was 1.27 days per year (*F*_1,15_ = 11.2, *P* = 0.0004, *R* ^2^ = 0.43). The hatching dates of Sanderlings did not change (*F*_1,16_ = 1.47, *P* = 0.24, *R* ^2^ = 0.07; Fig. 2B).

**Figure 3:**
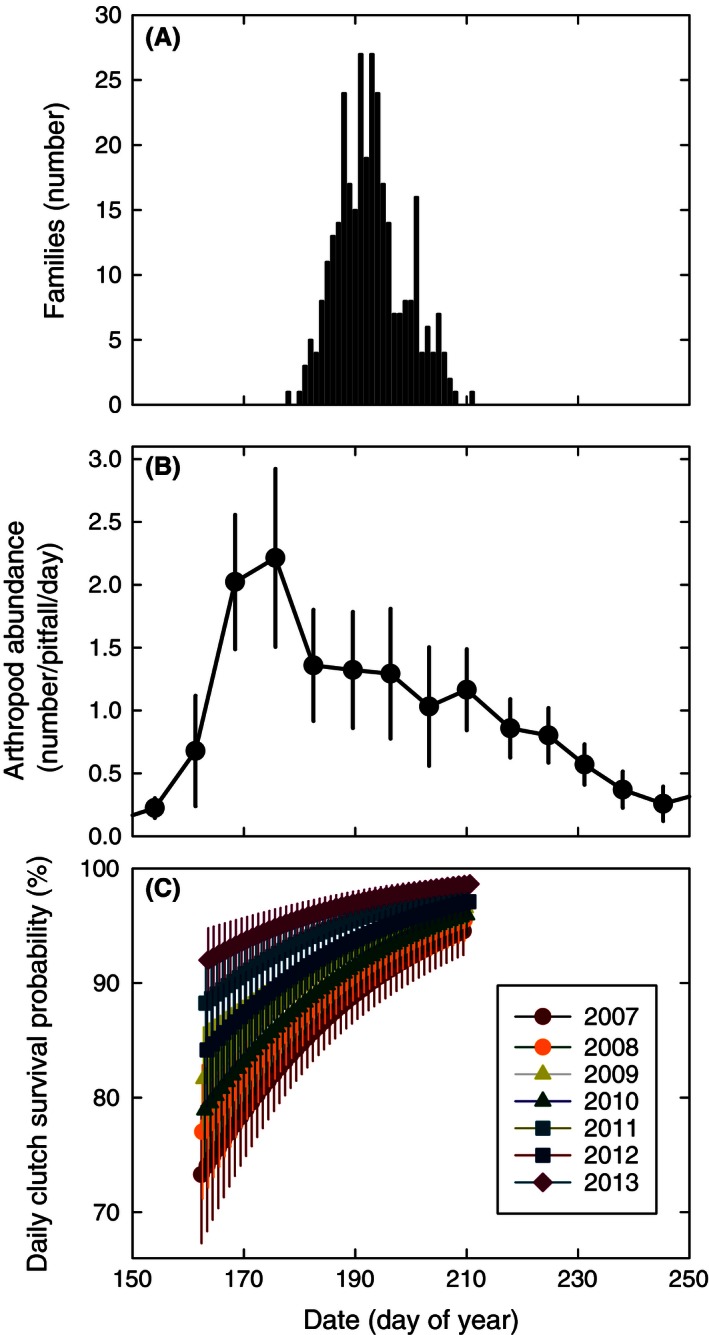
The frequency distribution of Sanderling (estimated and actual) hatch dates per day of year (A), seasonal changes in arthropod abundance (B), and the average seasonal increase in Sanderling clutch survival (C) in Zackenberg in 2007–2013. The average hatching date is 12 July (day of year = 193). The distribution in (A) includes failed clutches, whose hatch date was predicted based on egg flotation. Arthropod abundance (B) is expressed in number of individuals per pitfall trap corrected for the number of sampling days. The line shows the average ±SE across years. Samples on different dates were lumped within week. Seasonal patterns in daily clutch survival (C) of Sanderlings in Zackenberg in the years 2007–2013 based on the most parsimonious model in which year and date additively explain the variation in clutch survival.

**Figure 4:**
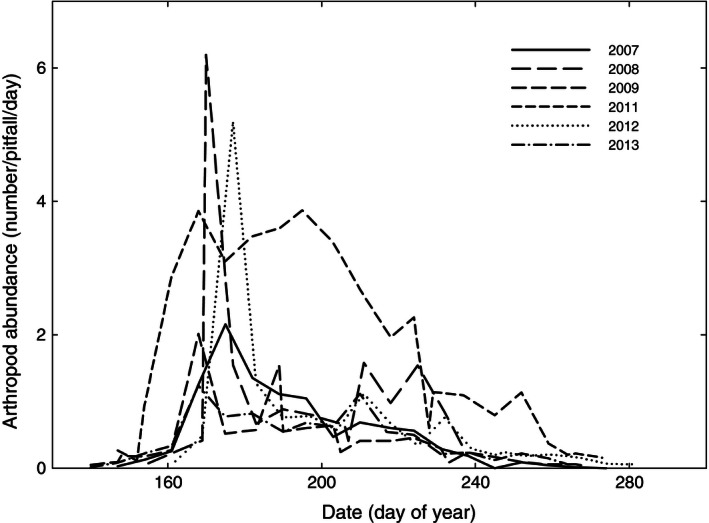
The seasonal changes in arthropod abundance in different summers (2007–2013).

Sanderling chicks hatched after the median peak abundance of prey in all but one of the years since 2000 (Fig. 2C). The phenological mismatch increased in the course of the study period but gradually leveled off after 2007 (*F*_2,14_ = 12.6, *P* < 0.001, *R* ^2^ = 0.64; Fig. 2C). In the last years of our study (2007–2013), Sanderlings hatched between 27 June and 30 July (average 12 July; Fig. 3A), which on average is 17 days after the median date of arthropod abundance (Fig. 3A,B). The temporal shape of the arthropod abundance differed considerably between years. In 2008 and 2012, the temporal abundance of arthropods was particularly high (with food peak heights of 6.2 and 5.2 arthropods pitfall^−1^·day^−1^, respectively) and narrow (with food peak widths of 39 and 36 days, respectively), whereas arthropods were particularly abundant in 2011 with almost the whole summer more arthropods in the pitfalls than at any date in the others years (food peak width of 40 days and food peak height of 3.9 arthropods pitfall^−1^·day^−1^; Fig. 4).

### Clutch survival

The model selection resulted in three competitive models with ΔAIC_c_ < 2 (Table 1) which all contain year and an additive date effect (date or snowdate), indicating that annual and intra‐annual ecological factors together explained most of the variation in clutch survival (Fig. 3C). The results of the second‐best model Year + Date^^2^ were similar to the most parsimonious model (Appendix S2).

**Table 1:** Summary of model selection results for clutch survival of Sanderling in Zackenberg 2007. A “*” indicates an interaction term and “^2” a quadratic term

Model	AICc	ΔAICc	*w*_*i*_	Model likelihood	*K*	Deviance
Year + Date	717.34	0	0.46	1.00	8	701.23
Year + Date^^2^	718.44	1.10	0.27	0.58	9	700.30
Year + Snowdate	718.68	1.34	0.24	0.51	8	702.57
Year + Date + Year*Date	714.40	7.06	0.01	0.03	14	696.08
Year	725.88	8.53	0.01	0.01	7	711.79
Date	726.27	8.93	0.01	0.01	2	722.26
Snowyear + Date	726.90	9.56	0.00	0.01	3	720.88
Date^^2^	726.93	9.59	0.00	0.01	3	720.90
Year + Snowdate + Year*Snowdate	727.36	10.02	0.00	0.01	14	699.05
Snowdate	729.81	12.47	0.00	0.00	2	725.81
Snowyear	735.71	18.37	0.00	0.00	2	731.70

Models are ranked by ascending ΔAICc, *w*_*i*_ is the model weight and *K* is the number of parameters.

Of the 164 Sanderling clutches in the entire study period that were depredated, the identity of the predator could be assessed in 58 cases (35%) only. Of those 58 clutches, 52 (90%) were taken by Arctic fox.

### Chick growth

**Figure 5:**
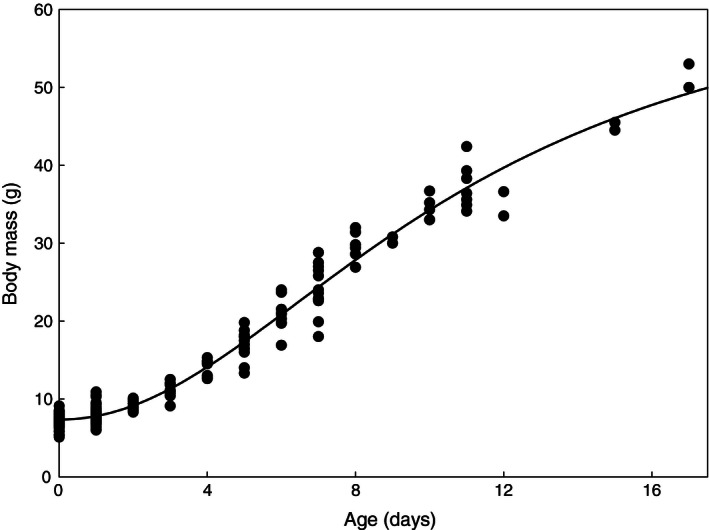
Growth of body mass of Sanderling chicks. Each dot represents the body mass of individual chicks at a given age (in days). Some dots overlap and some individuals were repeatedly measured. We accounted for pseudo‐replication in fitting the depicted curve, using a nonlinear mixed model with individual as a random factor (see Materials and Methods).

The body mass was determined for 310 chicks of known age of which 261 (84%) were from Zackenberg and the other 49 (16%) from Hochstetter Forland. Forty of these chicks (13%) were captured again before fledging. The logistic growth model (Fig. 5) described growth better than the Gompertz growth model (AIC_c_ = 1141 vs. 1158). The random effects (family and year) did not significantly improve the fit of our models. The AIC values of the model including all interaction terms and one or both random effects ranged from 399.2 (family as single random effect) to 403.9 (year as single random effect), whereas excluding any random effects resulted in a model with an AIC of 396.1. We therefore proceeded with a general linear model without random effects. After stepwise deletion of nonsignificant interaction terms and main effects (if not part of a significant interaction), our selected model indicated a significant effect of the interaction between food peak width and food peak height (*t*_3,50_ = 3.51, *P* = 0.001) and of the food peak width (*t*_3,50_ = 2.98, *P* = 0.004), but not of food peak height (*t*_3,50_ = 1.13, *P* = 0.134).

## Discussion

During a period of rapid climate warming and increasingly early snow melt in northeast Greenland, Sanderlings, unlike some other Arctic shorebirds (Liebezeit et al. 2014), did not advance the timing of breeding even though the median date of prey abundance was increasingly early. As a consequence, Sanderling chicks hatched after the peak abundance of arthropod prey in all but one of the 13 recent years. The advancement in the phenology of arthropod prey in northeast Greenland of 1.27 days per year was considerably faster compared to those in 18 studies in temperate areas in which the food peak advanced with only 0.19–0.87 days per year (Visser et al. 2012). In all of these 18 studies, those populations that did not track the advancement of their prey, experienced consequent negative fitness effects (Visser et al. 2012). Negative fitness effects of phenological mismatches were thus also to be expected in high Arctic Sanderlings and other Arctic shorebirds.

In our study, however, we have shown that the width of the food peak and the interaction with its height, but not the degree of phenological mismatch, positively affected chick growth. This suggests that food was still sufficiently abundant after it peaked (Durant et al. 2005; Miller‐Rushing et al. 2010) and that even chicks that hatched late after the median date of arthropod abundance, encountered sufficient food for normal growth. Given the low density of birds and the large amount of arthropods in northeast Greenland (Meltofte et al. 2007b), food abundance does not seem to limit chick growth per se, despite the strongly advanced arthropod emergence. A similar pattern has been observed in a population of Hudsonian Godwits (*Limosa haemastica* Linnaeus 1758) in subarctic Canada (Senner et al. 2016).We can however not rule out that high Arctic shorebirds optimize the timing of arrival and onset of reproduction in relation to the demanding prelaying period (Meltofte et al. 2007a) or the energetically challenging period of incubation (Piersma et al. 2003). If the fitness costs of arriving in a period with low food abundance and/or high energetic costs are larger than the costs of chicks hatching out of synchrony with their prey, no strong selection for a phenological match between food abundance and hatch date would be expected.

Schmidt et al. (2015) suggested that clutch predation may prevent phenological shifts in response to climate change. More recently, Harts et al. (2016) suggested that a higher predation risk on early arriving individuals may select for later arrival times of migratory birds. Indeed, birds may not only adjust their phenology to stay in pace with the timing of their prey, but also to escape their predators (Both et al. 2009). In our study, early laid Sanderling clutches had a larger risk to fail due to predation than later clutches. Yet, given the lack of negative fitness consequences of late hatching chicks, natural selection will not favor earlier laying. In most cases however, it was unknown whether Sanderling clutches were replacement clutches after an earlier clutch had been depredated. If early depredation causes birds to more often lay a replacement clutch, this will also result in later average hatch dates relative to the food peak.

The observed seasonal increase in clutch survival may be explained by several factors acting in concert. The limited search area for predators early in the season, when the tundra in most years is still covered by snow, combined with the largely synchronized laying dates of arriving birds, will probably make searches for bird eggs especially profitable early in the season (Byrkjedal 1980). Indeed, we showed that with a decreasing snow cover, daily clutch survival increased, although the effect of snow cover could not be distinguished from a general date effect. The date of 50% snow cover was increasingly early during our study and advanced faster than the median date of arthropod abundance and hatch. If these trends would continue, it would weaken the constraint to breed earlier because the period with early high risk of clutch predation, which is presumed to be related to snow cover, would then occur before the best period to breed in order chicks to hatch during the period of maximum food abundance.

Clutch predation and the successful hatching of clutches will lower the density of clutches in the course of the Arctic summer during which there is a date limit to when replacement clutches can be laid (Gates et al. 2013; Reneerkens et al. 2014). In combination with the increasing area where nests may occur as the snow melts, this will decrease the probability of predators to encounter clutches within a given period of time. It will depend on the local predator–prey interactions whether the risk of clutch predation is highest early in the season (as we report here), or in mid‐season (as reported by Smith and Wilson 2010).

Empirical studies on the timing of breeding of migratory birds have mainly focused on the synchrony with the peak in food availability without considering the threshold below which food abundance limits chick growth or survival. Also, the possible role of bird and egg predators has not received much attention. Indeed, much of our current knowledge on the effects of mismatches between avian predators and their prey comes from relatively few, long‐term studies on nest box breeding species (e.g., Winkler et al. 2002; Both et al. 2006; Charmantier et al. 2008; Dunn and Winkler 2010; Reed et al. 2013; Vedder et al. 2013) whose clutches are unnaturally protected against most predators. To understand how climate change affects ecosystems, we need to consider the full extent of trophic interactions (Ovaskainen et al. 2013) across all trophic levels (e.g., Both et al. 2009).

## Data Accessibility

Data used in this study are available from the Dryad Digital Repository: http://dx.doi.org/10.5061/dryad.3dk6r. Climate and arthropod data from Zackenberg are available at http://data.g-e-m.dk.

## Conflict of Interest

None declared.

## Supporting information


**Appendix S1.** Information about the relationship between daily arthropod numbers and estimates of total daily arthropod dry mass based on five additional pitfall transects in Zackenberg in 2007.
**Table S1.** Length to biomass equations for each family by size class where necessary.
**Figure S1.** The number of arthropod specimens in the pitfalls per day was positively correlated with the total estimated biomass (dry mass) on those days (linear regression *F*_1,44_ = 127, *P* < 0.0001, $R_{\mathrm{adj}}^{2}$ = 0.74).

**Appendix S2.**
**Figure S2.**
Model output of the second most parsimonious model explaining daily Sanderling clutch survival with year and an additive quadratic effect of date as explaining factors.
