# Supplementary material for: Effects of food abundance and early clutch predation on reproductive timing in a high Arctic shorebird exposed to advancements in arthropod abundance
Source: Ecol Evol. 2016 Sep 23;6(20):7375–86. doi: 10.1002/ece3.2361 (PMC5513252; doi:10.1002/ece3.2361)
Supplement: Supplementary file 1 — Appendix S1. Information about the relationship between daily arthropod numbers and estimates of total daily arthropod dry mass based on five additional pitfall transects in Zackenberg in 2007. Table S1. Length to biomass equations for each family by size class where necessary. Figure S1. The number of arthropod specimens in the pitfalls per day was positively correlated with the total estimated biomass (dry mass) on those days (linear regression F 1,44 = 127, P < 0.0001, Radj2 = 0.74). [file ECE3-6-7375-s001.docx]

**Supporting material S1**

In 2007, we installed five transects with 10 pitfalls each in Zackenberg to collect arthropods (Reneerkens *et al.* 2011). The pitfalls were emptied daily and all collected specimens were identified and measured. We used those data to study the strength of the correlation between number of arthropods and their total biomass estimate (daily totals for all pitfalls combined). The used equations of length to dry weight are those used by McKinnon *et al.* (2012) or, whenever possible, constructed by ourselves based on group-specific subsamples. All arthropod families are known to be part of Sanderling’s diet in northeast Greenland (Wirta *et al.* 2015).

**Table S1.** Length to biomass equations for each family by size class where necessary.

| Family | Size category | Equation | Reference |
| --- | --- | --- | --- |
| Calliphoridae |  | (exp(-3.374)*(L^2.158))*N | Sample et al. 1993 |
| Chironomidae | 0-4 mm | 0.049*N | Picotin 2008 |
| Chironomidae | 4-10 mm | 0.24*N | Picotin 2008 |
| Culicidae |  | 0.67*N | Picotin 2008 |
| Gnaphosidae |  | (-0.0017 + 1.2327*10^-6^ *exp(1.3726 *L))*N | Authors*^a^* |
| Hemiptera |  | (exp(-0.033+0.59029*L+(0.13674*L^2)))*N | Sage 1982 |
| Lepidoptera larvae |  | (-0.009 + 0.0038*exp(0.1073 *L))*N | Authors |
| Linyphiidae |  | (-0.0096+ 0.0081*exp(0.0975*L))*N | Authors*^b^* |
| Lycosidae |  | (-0.0096+ 0.0081*exp(0.0975*L))*N | Authors |
| Muscidae |  | (-0.002+ 0.0011*exp(0.1937*L))*N | Authors |
| Nymphalidae larvae |  | (0.0011*exp(0.1495*L))*N | McKinnon et al. 2012 |
| Noctuidae larvae |  | (0.0011*exp(0.1495*L))*N | McKinnon et al. 2012 |
| Pieridae larvae |  | (0.0011*exp(0.1495*L))*N | McKinnon et al. 2012 |
| Parasitica |  | (-0.0033+ 0.0018*exp(0.1577*L))*N | Authors |
| Phoridae |  | exp(-0.8503+0.69325*L +(-0.01762*L^2)*N | Sage 1982 |
| Scatophagidae | 0-5 mm | 0.53*N | Picotin 2008 |
| Scatophagidae | 5-10 mm | 0.98*N | Picotin 2008 |
| Sciaridae |  | 0.066*N | Picotin 2008 |
| Syrphidae |  | (exp(-8.503+0.6935*L+(-0.017613*L^2^)))*N | Sage 1982 |
| Tipulidae |  | (0.0008 + 0.0003 *exp(0.2055 *L))*N | Authors |
| Thomisidae |  | (-0.0017 + 1.2327*10^-6^ *exp(1.3726 *L))*N | Authors |
| Trichoceridae |  | 0.27*N | Picotin 2008 |

**NOTE:** In each equation, N is the number of individuals and L is the length of individuals.

*^a^*Equation for Thomisidae was used.

*^b^*Equation for Lycosidae was used.

**References**

Hódar, J.A. (1996) The use of regression equations for estimation of arthropod biomass in

ecological studies. *Acta Oecologica*, **17,** 421–433.

McKinnon, L., Picotin, M., Bolduc, E., Juillet, C. & Bêty, J (2012) Timing of breeding, peak

food availability, and effects of mismatch on chick growth in birds nesting in the High Arctic. *Canadian Journal of Zoology*, **90,** 961–971.

Picotin, M. (2008) Variation climatique, abondance d'arthropodes et phénologie de la

reproduction chez deux espèces de limicoles nichant dans le Haut Arctique. Masters degree thesis, Département de biologie, Université du Québec à Rimouski, Rimouski.

Reneerkens, J., Grond, K., Schekkerman, H., Tulp, I. & Piersma T (2011) Do uniparental

sanderlings *Calidris alba* increase egg heat input to compensate for low nest attentiveness? *PLoS ONE*, **6,** e16834.

Sample, B.E., Cooper, R.J., Greer, R.D., & Whitmore, R.C. (1993) Estimation of insect

biomass by length and width. *American Midland Naturalist*. **129,** 234–240.

Wirta, H., Vesterinen, E., Hambäck, P., Weingartner, E., Rasmussen, C., Reneerkens, J.,

Schmidt, N.M., Gilg, O. & Roslin, T. (2015) Exposing the structure of an Arctic food web. *Ecology and Evolution*, **5**, 3842–3856.

**Figure S1.** The number of arthropod specimens in the pitfalls per day was positively correlated with the total estimated biomass (dry mass) on those days (linear regression F_1,44_ = 127, P < 0.0001, R_adj_^2^ = 0.74).
