# Supplementary material for: Effects of food abundance and early clutch predation on reproductive timing in a high Arctic shorebird exposed to advancements in arthropod abundance
Source: Ecol Evol. 2016 Sep 23;6(20):7375–86. doi: 10.1002/ece3.2361 (PMC5513252; doi:10.1002/ece3.2361)
Supplement: Supplementary file 2 — Appendix S2. Figure S2. Model output of the second most parsimonious model explaining daily Sanderling clutch survival with year and an additive quadratic effect of date as explaining factors. [file ECE3-6-7375-s002.docx]

**Supporting material S2**

**Figure S2**. Model output of the second most parsimonious model explaining daily sanderling clutch survival with year and an additive quadratic effect of date as explaining factors.
